# The Need for Early Referral: Characteristics of Children and Adolescents Who Are Overweight and Obese Attending a Multidisciplinary Weight Management Service

**DOI:** 10.3390/children4110093

**Published:** 2017-10-31

**Authors:** Jacqueline L. Walker, Rebecca Malley, Robyn Littlewood, Sandra Capra

**Affiliations:** 1 School of Human Movement and Nutrition Sciences, The University of Queensland, Brisbane, QLD 4072, Australia; rebecca.malley@uqconnect.edu.au (R.M.); robyn.littlewood@health.qld.gov.au (R.L.); s.capra@uq.edu.au (S.C.); 2 Department of Dietetics and Food Services, Lady Cilento Children’s Hospital, Children’s Health Queensland Hospital and Health Service, Brisbane, QLD 4101, Australia; 3 Centre for Children’s Health Research, Children’s Health Queensland Hospital and Health Service, Brisbane, QLD 4101, Australia; 4 Statewide Queensland Child and Youth Clinical Network, Office of Strategy Management, Queensland Health, Brisbane, QLD 4000, Australia

**Keywords:** obesity, overweight, pediatric, multidisciplinary, weight management

## Abstract

There is a need to examine the issue of childhood obesity from a systems perspective. This study aimed to describe the baseline characteristics of children attending pediatric multidisciplinary weight management services and understand how this information will inform future service delivery. A total of 51 children and adolescents who were overweight and obese (27 male) and aged between two and 16 years participated. Body size measures such as body mass index (BMI) and body fat percentage were collected. Participants and their parents/guardians also completed questionnaires on dietary intake, behaviors and habits, physical activity and health-related quality of life. A total of 72% of participants were classified as morbidly obese. Adolescents had significantly lower scores for overall diet, physical activity and particular health-related quality of life scores. No significant correlations were found between BMI z-scores and diet, physical activity and health-related quality of life. In adolescents, correlations were detected between dietary scores and health-related quality of life. Results confirm the need to critically examine the current context to adapt and tailor interventions to individual circumstances, and when combined with focused referral, triaging and screening processes, should assist in delivering the right care at the right time.

## 1. Introduction

There is a clear need to understand the issue of childhood obesity from a systems perspective. Overweight and obesity in childhood and adolescence remains a critically important health care issue, both in Australia and internationally [1,2]. Obesity is a complex health issue, with many social, environmental, biological, and individual factors contributing to weight gain across the life span [3]. Approximately one in four Australian children aged five to 17 years (27.4%) are classified as overweight or obese [1], which is similar to the average rate for the countries that form the Organization for Economic Co-Operation and Development (OECD), at approximately 23% [4]. While there appears to be no change in the proportion of children who were overweight or obese since 2011–2012 [1], the associated health risks remain considerable. Pediatric overweight and obesity can significantly affect a child’s psychological wellbeing, self-esteem, quality of life and self-image [5]. In addition, the risk that obesity continues on into adulthood, with concomitant complications such as hypertension, type 2 diabetes mellitus, and cardiovascular complications, can have a significant impact on overall health and wellbeing [2]. It also places considerable burden on the public health care system in terms of healthcare costs [6]. Overall, the complexity of the issue requires acknowledgement, rather than trivialization or simplification, and coordination of interventions at all levels of a system in order to significantly impact the prevalence of childhood obesity [7].

Current literature highlights the need for a systems approach to the issue of childhood obesity. The World Health Organization (WHO) Commission on Ending Childhood Obesity recently released a report which details actions required to address the issue of childhood obesity worldwide [8]. The plan acknowledges that while there is no single solution to the problem, stakeholders from relevant government departments and public and private organizations need to work together to achieve positive outcomes for populations of children and adolescents who are overweight and obese. Guiding principles emphasize whole-of-government and whole-of-society approaches, rather than short term programs, with key elements to ensure successful implementation including prioritization; awareness, communication and education; mobilization of resources; and capacity building [8]. This plan sounds promising, however, the majority of one-off programs aimed at preventing or managing childhood obesity have universally failed, as there is usually a lack of integration within the health care system [6,9]. What is now required is a greater understanding of how researchers, clinicians and stakeholders can effect change within the system, and effectively integrate a variety of strategies that, when combined within the system, address the issue of childhood overweight and obesity.

Health services research, with a focus on implementation science, is a systems approach that enables researchers to understand the strengths and weaknesses of a health care system, and how research findings and evidence can be translated and integrated into changes in health care policy and practice [10,11]. More specifically, “health services research is the multidisciplinary field of scientific investigation that studies how social factors, financing systems, organizational structures and processes, health technologies, and personal behaviors affect access to health care, the quality and cost of health care, and ultimately our health and well-being. Its research domains are individuals, families, organizations, institutions, communities, and populations.” [12]. By taking a pragmatic approach to the issue, researchers are able to answers questions that are relevant in real-life settings, and have the potential to impact significantly on health care practices and service delivery [7]. The evidence-base for multicomponent lifestyle interventions for the management of pediatric overweight and obesity is clear, and numerous clinical practice guidelines have been published [3,13,14], however there continues to be ongoing inconsistencies in the translation of this evidence into practice, alongside a limited understanding of how to achieve change within the system. Some of the major challenges to organizational change is shifting organizational culture, and providing the real-world, contextual evidence that a significant system problem exists [7]. This evidence is critical in order to gain the necessary buy-in from stakeholders needed for an effective change in clinical practices [7].

There are currently limited public health services in Australia for children who are overweight or obese, and these services are not always delivered in a consistent, integrated way. The majority of children do not receive evidence-based care, despite our knowledge of what works [15]. To investigate this issue further, the authors developed a research agenda focused on using systems thinking and a pragmatic, action research design, to examine the approach to pediatric weight management services in Queensland, Australia, and identify leverage points for change. The first step in this process was the expansion and evaluation of pilot multidisciplinary weight management clinics to treat those children and adolescents who were already classified as overweight or obese and referred for services. The design included investigating the baseline characteristics of the children and adolescents attending these multidisciplinary services to understand what is happening from a systems perspective, in terms of how and who is referred for services and at what stage in terms of overweight and obesity. This will inform researchers and clinicians on what this population looks like, understand how referral processes are used within the system, and how this can help us to provide clearer direction for services, modify clinical practices, up-skill staff, assess effectiveness and design more appropriate interventions. In turn, it is hoped that this will reduce attrition rates and contribute to a sustainable service. The aim of this study, therefore, was to describe the baseline characteristics of children attending a pediatric multidisciplinary weight management clinic over a one-year time frame at a tertiary hospital and satellite site in Queensland, Australia, in the context of health services implementation research.

## 2. Materials and Methods

This prospective cross-sectional study was part of a larger project investigating the expansion, evaluation and sustainability of a public multidisciplinary pediatric weight management service in Queensland, Australia. Ethics approval was obtained from the Human Research Ethics Committees at both Children’s Health Queensland Hospital and Health Service (CHQ HHS) and Bellberry Limited respectively. The study is registered under the Australian New Zealand Clinical Trials Registry (ID: ACTRN12616000101482) and is a pragmatic research trial. Written informed consent was provided by all parents/guardians and obtained from participants where appropriate. Data collection occurred between June 2016 and June 2017 at two clinic locations: Lady Cilento Children’s Hospital (LCCH), a large central service and at a satellite site, The University of Queensland (UQ) Ipswich Nutrition and Dietetics practice (IPSWICH), both situated in Queensland, Australia. All children and their parents/guardians attending the clinic were invited to participate in the study. Inclusion criteria for acceptance in the clinics were the following: (i) referred by a pediatrician, primary care provider (PCP), member of the education department or allied health and aged between 0 and 18 years; and (ii) participants less than two years of age were required to be above the 97th percentile on the WHO weight-for-age growth charts [16] and gaining weight rapidly, or been assessed by a pediatrician or PCP as having weight concerns and above the healthy weight range; participants older than two years of age were required to have a body mass index (BMI) >85th percentile on the United States Centers for Disease Control and Prevention (US-CDC) charts [17] or the WHO charts [18]. Referral guidelines to the clinic were based on National Health and Medical Research Council (NHMRC) Clinical Practice Guidelines for the Management of Overweight and Obesity in Adults, Adolescents and Children in Australia [3]. Children with serious co-morbidities requiring urgent weight management or a suspected underlying metabolic or endocrine cause were eligible to participate in the study. Families who could not understand English well, and where an interpreter was not available were excluded from the study. The research data, including demographic information, was collected either immediately after the first clinic attendance, or at a separate appointment that was scheduled within two weeks of the first attendance.

The multidisciplinary weight management clinic offered the same service to children, adolescents and families across the two sites, and involved a medical practitioner (pediatrician or endocrinologist), dietitian and psychologist. It was an intensive, clinical management program in a real-life setting, and all advice and guidance provided was individualized and evidence-based, following recommendations from the Clinical Practice Guidelines for the Management of Overweight and Obesity in Adults, Adolescents and Children in Australia [3]. Advice and guidance included areas such as diet modification, nutrition counselling, promotion of an active lifestyle, behavior modification strategies, supportive parental involvement, and a comprehensive medical evaluation.

### 2.1. Anthropometric Measures

Height was measured to the nearest 0.1 cm using a stadiometer (Seca Model 213 or 264, Seca, Hamburg, Germany) and weight to the nearest 0.1 kg was collected using digital scales (Seca Medical Scales Model 876, Seca, Hamburg, Germany) with participants wearing light clothing and no shoes. BMI (kg/m^2^) was calculated using the above information and BMI z-score for age and gender was determined via US-CDC or WHO z-score data files [16,17,18]. The International Obesity Taskforce (IOTF) classifications were used to categories BMI [19]. A measure of percentage body fat was collected using foot-to-foot bioelectrical impedance analysis (BIA) scales.

### 2.2. Dietary Intake, Behaviours and Habits

All parents/guardians or participants, depending on age and capacity, completed a paper-based version of the E-KINDEX (Electronic Kids Dietary Index) questionnaire [20]. This tool was selected to assess a child’s obesogenic habits, and contains three sections; dietary intake, dietary behaviors and eating beliefs, and dietary practices or habits. The overall maximum score is 87 with higher scores indicating a healthier food intake as well as behaviors and habits associated with a lower BMI [20].

### 2.3. Physical Activity

Physical activity (PA) was assessed via paper-based questionnaires and completed by participants or parent/guardian depending on age and capacity. The validated pre-school age physical activity questionnaire (Pre-PAQ) was used to assess young children who were not yet of school age [21]. For this study, only question 34 and 37 were completed, as these are the only scored questions, which have been previously validated against accelerometer data at the group level [21]. The other questions provide demographic, contextual and parental PA information which had either been collected, or was not of interest to our study. Scoring of questions 34 and 37 were reported as total minutes of Level 3 (light activity) to Level 5 (moderate to vigorous) per day [21]. The physical activity questionnaire for older children (PAQ-C) and physical activity questionnaire for adolescents (PAQ-A) were used for participants in primary school (approximate age 6–13 years) and high school (approximate age 13–17 years) respectively [22,23]. These valid and reliable questionnaires provide a general measure of physical activity and generate an overall score from one to five, with one indicating low PA, and five indicating high PA [22,23].

### 2.4. Health-Related Quality of Life

Health-related Quality of Life (HRQOL) was measured using the paper-based PedsQL™ Measurement Model for the Pediatric Quality of Life Inventory™ questionnaire, validated for ages 2–18 years [24]. PedsQL™ has separate questionnaires for different age groups (2–4 years, 5–7 years, 8–12 years and 13–18 years), with a parent proxy and child version for each age group with the exception of children aged 2–4 years. Each parent/guardian and the participant (depending on age and capacity) completed the questionnaire separately. The questionnaire is separated into four sections: physical, emotional, social and school functioning. From this, three scores are generated; physical, social (emotional, social and school sections) and an overall score. The maximum for each score is 100, with a higher score representing a higher HRQOL [24].

### 2.5. Statistical Analyses

Statistical analyses were performed using Statistical Package for the Social Sciences version 23 (IBM SPSS Statistics 23.0). Standard diagnostics tests such as normality and homogeneity of variance were performed. All tests were two tailed and statistical significance was set at an α value of 0.05 apart from Mann-Whitney U tests to identify differences between the three age groups and here it was set at 0.017 to avoid Type 1 error. Participants were grouped according to age: 0–4.99 years (young children), 5–11.99 years (children) and 12–17.99 years (adolescents) and descriptive statistics determined. Differences in variables between groups were assessed using either one-way ANOVA with the post-hoc Tukey tests, Kruskal Wallis tests with post-hoc Mann-Whitney U tests, *t*-tests and Mann-Whitney U tests (for variables compared across two age groups only) or via ANCOVA with post-hoc multiple comparisons, adjusted for the Index of Relative Socio-Economic Disadvantage Score (IRSD). Differences between child-reported and parent proxy-reported quality of life scores were calculated using paired samples *t*-tests and Wilcoxon signed rank test. Relationships between key variables were assessed using partial correlation, controlling for the effect of IRSD scores.

## 3. Results

Fifty-one children and adolescents (53% male) ranging in age from 1.6 years to 16.3 years participated in the study over a one-year period (July 2016 to June 2017). At the time of assessment, 53% of the participants attended the LCCH clinic, and 47% attended the IPSWICH clinic. Based on the IOTF classification for BMI for age and gender, 14% (*n* = 7) were classified as overweight, 14% (*n* = 7) as obese and 72% (*n* = 37) as morbidly obese [19]. A total of 28 participants (55%) lived in areas that indicate relatively greater disadvantage according to the IRSD score [25]. Medical specialists within the LCCH were the most frequent referral source (*n* = 18 participants, 35%), followed by paediatricians within the LCCH (*n* = 9, 17.5%), external general practitioners (*n* = 7, 14%), external dietitians (*n* = 7, 14%), obesity specialists within the LCCH (*n* = 6, 11.5%) and external paediatricians (*n* = 4, 8%). All participants were either Anglo-Celtic or East Asian in ethnicity.

When grouping participants according to age, 12% (*n* = 6) were aged between 0 and 4.99 years (young children), 45% (*n* = 23) were aged between 5.00 and 11.99 years (children), and 43% (*n* = 22) were aged between 12.00 and 17.99 years (adolescents). Comparisons were made between the three age groups for anthropometric variables (Table 1), and dietary, PA and HRQOL results (Table 2). Young children had the highest BMI z-scores of all participants (*p* < 0.006), and there were no significant differences between BMI z-scores for children and adolescents. No differences in body fat percentage were found between children and adolescents, and median body fat percentage results for both groups were considerably higher than the 98th centile for all ages and genders on current reference curves [26]. Body fat percentage was not able to be measured for all young children due to difficulties in standing still for assessment (Table 1).

When considering the overall dietary score, adolescents scored significantly lower compared to children (mean difference (MD) = 6.6, 95% confidence of interval (CI) = 0.24–13.0, *p* = 0.039). Young children had the highest scores for dietary behaviors and eating beliefs, significantly greater than scores for adolescents (MD = 5.7, 95% CI = 0.94–10.47, *p* = 0.014). Dietary intake scores were similar across all age groups (Table 2).

Comparisons for PA could only be made between the children and adolescents, due to the different questionnaire used for the young children. Adolescents had significantly lower PA levels when compared to children (MD = 0.48, 95% CI = 0.1–0.86, *p* = 0.014) (Table 2).

Results regarding HRQOL showed that parent proxy-reported social HRQOL was significantly lower for adolescents when compared to young children (MD = 23.70, 95% CI = 2.30–45.08). No other significant differences were found between the age groups for child or parent proxy-reported HRQOL. When comparing the child and parent proxy-reported HRQOL scores within groups, parent scores were generally lower overall and for each section. For the children, parent proxy-reported HRQOL was lower for overall (f (20) = 2.75, *p* < 0.05) and social scores (z = −2.016, *p* < 0.05). Within the adolescents, parent proxy-reported HRQOL was lower overall (z = −2.25, *p* < 0.05) and for both physical (z = −2.23, *p* < 0.05) and social scores (f (18) = 2.55, *p* < 0.05) (Table 2).

BMI z-score was compared to HRQOL, dietary overall and PA scores across age groups, but no obvious significant relationships were found. The relationship between HRQOL and dietary measures was investigated, with significant findings in the adolescent age group (Table 3). A strong, positive correlation was evident between adolescent-reported overall HRQOL and overall diet score (r = 0.709, *p* < 0.01), dietary intake score (r = 0.604, *p* < 0.01) and dietary behaviors and eating beliefs score (r = 0.496, *p* < 0.05). A medium to strong correlation was found between parent proxy-reported HRQOL and dietary practices or habits score (r = 0.541, *p* < 0.05) in the adolescent aged group. No relationships between HRQOL and dietary measures were evident in the children and young children age groups.

## 4. Discussion

The aim of this study was to describe the baseline characteristics of children attending a pediatric multidisciplinary weight management clinic in Queensland, Australia, in the context of health services implementation research. The authors wanted to understand, at the systems level, what strategies are working to support the prevention and management of pediatric overweight and obesity in Queensland, and what needs to change. Results showed that children entering the clinics were not assessed on parameters of diet, physical activity or HRQOL prior to referral, and were considerably overweight as per international definitions, with 72% of participants classified as morbidly obese. This may have been due, in part, to triaging practices within the system, where children classified as overweight were generally placed into a dietitian-only clinic, rather than a multidisciplinary clinic. These results may also reflect referring practitioner decisions, who may only be referring children and adolescents when their BMI is within the obese category and other concerns are apparently obvious. This is supported by the data which show that only 14% (*n* = 7) of children or adolescents were referred by an external general practitioner, compared to 65% (*n* = 33) referred by a specialist or pediatrician within the LCCH. Of these 33 children and adolescents, data regarding their contact with the public or private health care system was not collected, so it is unclear why concerns regarding weight were not flagged earlier. Ideally, this should be happening at the primary care level. Additionally, the sample size did not include children or adolescents of ethnicities considered to be at higher risk of overweight and obesity in Australia, such as Indigenous Australians or those from Maori and Pacific Islander cultures [27,28]. Ensuring timely identification, referral and management for these children and adolescents is critical, and needs to be investigated further from a systems-level.

Within this cross-sectional study, measures of diet, PA and aspects of HRQOL generally declined in the older age groups. Scores for overall diet, behavior and eating beliefs and practices or habits sections of the dietary questionnaire were significantly lower in adolescents, and we speculate that this could be related to increased exposure to discussions of weight, previous dieting attempts or engaging in more obesogenic habits such as skipping breakfast [29]. Dietary intake scores were fairly similar across the three age groups. This stability in scores may reflect the measurements within the tool, rather than the absence of any differences, as the tool measures types of food and the number of times the foods are consumed across a week, rather than quantity which is typically highest in adolescents [29].

PA was also significantly lower within the adolescent age group which matches trends found in national health surveys in Australia [30]. While scores were significantly different, both children and adolescents were still doing only low amounts of activity when compared to recommendations [3].

Outcomes concerning HRQOL showed that parents proxy-reported lower HRQOL scores when compared to reports by their children. Within the adolescent age group, dietary scores (including overall diet, dietary intake, and dietary behaviors and eating beliefs) increased as self-reported HRQOL scores increased, highlighting an important relationship that was not evident in the children in the study. This has been described previously in the literature in a large prospective study in Australia by Jacka et al. [31] who found a relationship between unhealthy dietary patterns and poorer mental health in adolescents—higher unhealthy diet scores at baseline predicted lower HRQOL scores (using the same tool as the current study) at follow up one year later, and improvements in diet quality were reflected by improvements in mental health [31]. These outcomes support the need for new triaging and screening options to identify those adolescents who would benefit from targeted, intensive psychological input.

The absence of any significant relationships between BMI z-score and diet, PA and HRQOL measures was unexpected. It was thought that these results may have provided an interesting insight into what to target in multicomponent interventions for specific age groups. These findings may be due to the fact, that 72% of the cohort were classified as morbidly obese, and the variation in BMI z-scores across all age groups was small.

Clearly, more research is required to understand why children and adolescents are not being identified and referred to services in a timely manner, and what can be done from a systems perspective to change this practice. This is important, given that recent evidence from similar multidisciplinary clinics in real-life settings on an international level suggests that treatment outcomes are better when children were younger [32] and that early intervention for weight management is preferred and considered more cost-effective [15]. Consideration also needs to be given to ensure that the most appropriate triaging guidelines are utilized once children and adolescents have been referred to services. Currently, triaging guidelines for the LCCH and IPSWICH clinics are generally based on weight and associated co-morbidities only. The results of this study, however, show that other factors such as age, diet, PA and/or HRQOL should also be considered. Screening for these factors during the triaging process can help determine the level of support and intensity required for patients, which will in turn contribute to an efficient use of services, which helps contain costs. Results also confirm that clinics cannot use a one-size-fits-all approach to treatment and management given the differences evident at commencement of the clinic between the groups of children. Services may need to consider if the formation of an adolescent-specific clinic, where psychological input occurs more frequently that for the younger children, would be a beneficial use of resources given their clinical and service context.

The main limitation of this study is the small sample size potentially causing insufficient power to detect correlations. The population of interest is difficult to recruit, and attrition is high, resulting in small sample sizes which may not be representative of the larger population of children and adolescents who are overweight or obese. Missing data was also a common theme as a result of unavoidable reasons such as child age or behavior. Another limitation was that outcome measures for dietary intake, behaviors and habits and physical activity were self-reported rather than objectively measured. Given the pragmatic nature of the study, and the limitations when embedding research into clinical practice, this was a necessary compromise. Furthermore, the E-KINDEX tool has, at this stage, only been validated for children aged 9 to 13 years [20]. Lastly, due to the choice of tools for physical activity, comparisons were unable to be made between young children and the other two age groups.

## 5. Conclusions

There is no single solution to the problem of pediatric overweight or obesity, however it remains a significant health problem and a focus on timely identification of concerns, appropriate referral systems, and dedicated services is critical. Results of this study found that adolescents are already experiencing detrimental consequences related to being overweight or obese, such as lower scores related to diet and dietary practices or habits, and lower levels of PA. Results identified that system referral processes are not effective, and confirm the need to critically examine the current context to further investigate these processes and adapt interventions to individual circumstances, supported by focused triaging and screening processes to enhance the opportunity for individual success. There is a clear need to implement these research findings for system-wide change to enable efficient, effective service delivery options at all levels of health care that can be up-scaled throughout the public health system.

## Figures and Tables

**Table 1:** Anthropometric characteristics of participants by age groups.

Age Group in Years (Sample Size)	Young Children: 0–4.99 Years Old (*n* = 6)	Children: 5–11.99 Years Old (*n* = 23)	Adolescents: 12–17.99 Years Old (*n* = 22)
Age (years)	3.0 ± 1.2 ^a,b^	8.8 ± 1.6 ^a^	13.7 ± 1.2
BMI z-score	3.2 (2.6, 5.9) ^a,b^	2.5 (1.4, 3.4)	2.4 (1.4, 2.8)
Weight (kg)	22.4 (14.6, 40.4) ^a,b^	56.4 (39.2, 101.5) ^a^	97.4 (52.3, 137.4)
Height (cm)	98.5 (80.5, 117.6) ^a,b^	142.9 (113.2, 159.5) ^a^	163.7 (136.2, 187.0)
Body fat (%)	-	38.1 ± 8.5	42.8 ± 5.8

Data presented as mean ± standard deviation (SD), or median (minimum, maximum); ^a^ significantly different from adolescents aged 12–17.99 years (*p* < 0.001); ^b^ significantly different from children aged 5–11.99 years (*p* < 0.006); via ANOVA with post-hoc Tukey tests (weight, body fat), or Kruskil Wallace and post-hoc Mann Whitney tests (age, body mass index (BMI) z-score, height).

**Table 2:** Dietary, physical activity and health-related quality of life scores by age groups.

Age Group in Years (Sample Size)	Young Children: 0–4.99 Years Old (*n* = 6)	Children: 5–11.99 Years Old (*n* = 23)	Adolescents: 12–17.99 Years Old (*n* = 22)
Dietary intake	24.5 (10, 28)	24.0 (16, 30)	21.5 (13, 32)
Dietary behaviors and eating beliefs	15.2 ± 4.1 ^a^	11.2 ± 3.6	9.2 ± 4.4
Dietary practices or habits	20.5 (12, 27)	22.0 (13, 25)	18.0 (12, 27)
Overall diet	58.5 ± 12.9	55.4 ± 6.1 ^a^	48.9 ± 9.2
Child HRQOL physical	-	75 (40.6, 98.75)	71.9 (34.4, 93.8)
Child HRQOL social	-	64.6 (15, 93.3)	60 (1.67, 86.7)
Child HRQOL overall	-	68.9 ± 16.5	60.1 ± 17.4
Parent HRQOL physical	64.0 (28.13, 100)	50 (0, 100)	53.1 (18.3, 87.5) ^b^
Parent HRQOL social	66.5 ± 7.9 ^a^	55.7 ± 18.3 ^b^	45.3 ± 18.8 ^b^
Parent HRQOL overall	65.2 (48.6, 82.1)	55.2 (20.7, 92.4) ^b^	47.3 (23.9, 73.9) ^b^
PA for older children and adolescents	-	2.5 ± 0.6 ^a^	2.1 ± 0.6
Pre-PAQ	1426 ± 945	-	0

Data presented as mean ± SD, or median (minimum, maximum); ^a^ significantly different from adolescents aged 12–17.99 years (*p* = 0.006) via ANCOVA with post-hoc multiple comparisons, adjusted for the Index of Relative Socio-Economic Disadvantage Score (IRSD); ^b^ significantly different from child-reported HRQOL (Health-related Quality of Life) scores (*p* < 0.05) via paired samples *t*-tests or Wilcoxon Signed-Rank Test. PA: physical activity; Pre-PAQ: pre-school age physical activity questionnaire.

**Table 3:** Correlations between health-related quality of life and dietary outcomes within each age group.

Age Group in Years (Sample Size)	Young Children: 0–4.99 Years Old (*n* = 6)	Children: 5–11.99 Years Old (*n* = 23)	Adolescents 12–17.99 Years Old (*n* = 22)
Child-reported HRQOL overall			
Overall diet	-	0.188	0.709 **
Dietary intake	-	−0.051	0.604 **
Dietary behaviors and eating beliefs	-	0.214	0.496 *
Dietary practices or habits	-	0.151	0.349
Parent proxy-reported HRQOL overall			
Overall diet	−0.688	0.372	0.434
Dietary intake	−0.798	0.074	0.302
Dietary behaviors and eating beliefs	−0.371	0.371	0.124
Dietary practices or habits	−0.536	0.189	0.541 *

* *p* < 0.05 (two tailed) via partial correlation, controlling for the effect of IRSD scores; ** *p* < 0.01 (two tailed) via partial correlation, controlling for the effect of IRSD scores.
